# Development of a biophysical screening model for gestational hypertensive diseases

**DOI:** 10.1186/s12929-019-0530-0

**Published:** 2019-05-20

**Authors:** Sharona Vonck, Anneleen S. Staelens, Dorien Lanssens, Kathleen Tomsin, Jolien Oben, Liesbeth Bruckers, Wilfried Gyselaers

**Affiliations:** 1 Faculty of Medicine and Life Sciences, Hasselt University, Agoralaan, 3590 Diepenbeek, Belgium; 2 Department of Obstetrics & Gynaecology, Ziekenhuis Oost-Limburg, Schiepse Bos 6, 3600 Genk, Belgium; 3 Department Physiology, Hasselt University, Agoralaan, 3590 Diepenbeek, Belgium; 4 Interuniversity Institute for Biostatistics and statistical Bioinformatics, Hasselt University, Agoralaan, 3590 Diepenbeek, Belgium

**Keywords:** Gestational hypertensive diseases, Hypertension, Preeclampsia, Screening, Biophysical parameters, Prediction

## Abstract

**Background:**

To investigate the possibility of using maternal biophysical parameters only in screening for the different types of gestational hypertensive diseases.

**Methods:**

A total of 969 pregnant women were randomly screened in first and second trimester, of which 8 developed Early-onset Preeclampsia, 29 Late-onset Preeclampsia, 35 Gestational Hypertension and 897 women had a normal outcome. An observational maternal hemodynamics assessment was done via standardized electrocardiogram-Doppler ultrasonography, Impedance Cardiography and bio-impedance, acquiring functional information on heart, arteries, veins and body fluid. Preliminary prediction models were developed to test the screening potential for early preeclampsia, late preeclampsia and gestational hypertension using a Partial Least Square Discriminant Analysis.

**Results:**

A combined model using maternal characteristics with cardiovascular parameters in first and second trimester offers high screening performance with Area Under the Curve of 99,9% for Early-onset Preeclampsia, 95,3% for Late-onset Preeclampsia and 94% for Gestational Hypertension.

**Conclusions:**

Using biophysical parameters as fundament for a new prediction model, without the need of biochemical parameters, seems feasible. However, validation in a large prospective study will reveal its true potential.

## Background

Gestational hypertensive disorders (GHD), including gestational hypertension (GH), preeclampsia and essential hypertension, contribute to maternal and perinatal mortality and morbidity, and are linked to health problems in later life [1]. Their aetiology is considered multifactorial, is still not completely understood and troubles prediction. Aside chronic hypertension, onset of GHD’s develop in the second half of pregnancy. However, a detection of subclinical signs in the first half of pregnancy opens perspectives for prophylactic treatment (low dose aspirin) or preventive measures (blood pressure recording) towards reduction of the adverse maternal and neonatal outcome [2].

Current screening programs are based on a combination of maternal characteristics (age, ethnicity, body mass index, nulliparity, family or personal history) with biophysical (mean arterial pressure, pulsatility index) and biochemical markers (soluble Fms-like tyrosine kinase-1 (sFlt-1), placental growth factor (PlGF), pregnancy-associated plasma protein A (PAPP-A)), offering reported detection rates of > 90% for early-onset preeclampsia (EPE) at 10% false positive ratio. However, for late-onset preeclampsia (LPE) or GH, those models are troubled with a rather poor performance (LPE: 35–76%; GH: 18–21%) [3–5]. Other efforts were done seeking other biomarkers [6], metabolites [7], aorta intima media thickness [1], flow mediated dilatation of the brachial artery [8] or proteomics [9], but neither of them were highly promising.

In hypertensive disorders, it is already shown that cardiovascular parameters differ from the first trimester onwards and that different sets of parameters relate to different types of hypertension [10, 11]. We aimed to investigate the strength of using these maternal “type-specific” biophysical parameters only as screening to predict the different types of gestational hypertensive diseases.

## Methods

### Definitions of gestational hypertensive disorders

Diagnoses were made during the course of the normal clinical care by the investigators according to the ISSHP guidelines [2] and based upon the patient’s clinical record. After birth, gestational outcome was defined and data were categorized in normotensive pregnancy (NP), GH, EPE, LPE, essential hypertension (EH) and all classified according to birth weight percentile ≤ or > 10% as small for gestational age (SGA) or non-SGA respectively. GH was defined as a non-proteinuric hypertension, developed after 20 weeks of gestation. Preeclampsia was defined as new-onset hypertension with proteinuria ≥300 mg/24 h, labelled as early-onset at clinical presentation of < 34 weeks and late-onset at presentation of ≥34 weeks of gestation. EH was a non-proteinuric hypertension with need for antihypertensive medication, developed < 20 weeks. GH, LPE and EPE were used for analysis (Appendix).

### Study design

Approval of the Ethical Committee was obtained before study onset (MEC ZOL, reference: 13/090 U) and informed consent before inclusion. One group of inclusions were normotensive women with singleton pregnancies, between 8^+ 0^ - 15^+ 6^ weeks of gestation, presenting at the obstetric ultrasound scanning clinic at Ziekenhuis Oost-Limburg Genk for their routine first trimester ultrasound scan who were invited to participate in an observational study between 2011 and 2016 where a cardiovascular assessment was performed. The second group were women with established risk factors, such as concommittant disease, obstetric or medical history, referred by their gynaecologist to our maternal-fetal medicine unit. The cardiovascular assessment was repeated between 18^+ 0^ - 27^+ 6^ weeks of gestation in 64% of the patients. The other 36% of patients were lost to follow-up for logistical reasons or delivered elsewhere. At delivery, all included pregnancies were categorized according to confirmed and documented outcome. For this preliminary analysis, we included patients with NP, GH and LPE diagnoses, all without SGA neonates to obtain unbiased groups with a pure hemodynamic profile (Appendix). As exception, the group of EPE patients were included with and without SGA neonates, partly from the theoretical concept that poor foetal growth is an intrinsic feature of EPE and partly to increase the numbers of pathological cases as they were otherwise too low (Appendix). Pregnancies complicated with EH (with or without SGA, *n* = 28), superimposed late preeclampsia with or without SGA (*n* = 5), HELLP with or without SGA (*n* = 3) and isolated SGA (*n* = 92) were excluded from the prediction model analysis, as well as multiplet pregnancies (n = 2) (Appendix).

### Maternal characteristics

Maternal characteristics were obtained through history taking and retrospective review of the medical records for each patient. This included maternal age, pregestational weight, height, pregestational body mass index (BMI), allergy, parity, history on diabetes/intra-uterine death/intra-uterine growth restriction/thrombophilia/ hypertension.

### Cardiovascular profile

A maternal cardiovascular profile was taken in every pregnant woman combining three non-invasive techniques (Impedance cardiography, ECG-Doppler ultrasound and bio-impedance) to obtain functional information about the complete maternal circulation (arteries, veins, heart, and body fluid content) (Table 1). A standardized protocol, as described below, was used as reported in previous other studies [12–14].

**Table 1:** Overview of all parameters derived in one cardiovascular assessment session with the three techniques

	ECG-Doppler	ICG	Bio-impedance
Heart		Heart Rate (HR) Stroke Volume (SV) Cardiac Output (CO) Pre-ejection Period (PEP) Left Ventricular Ejection Time (LVET)	
Arteries	Left + Right Arterial Pulse Transit (APT) Left + Right Pulsatility Index (PI) Left + Right Resistivity Index (RI)	Velocity Index (VI) Acceleration Index (ACI) Total Peripheral Resistance (TPR) Diastolic Blood Pressure (DBP) Mean Arterial Pressure (MAP)	
Veins	Hepatic Venous Pulse Transit (VPT) Left and Right Renal VPT Hepatic Vein Index (HVI) Renal Interlobal Vein Index (RIVI)		
Fluid			Total Body Water (TBW) Extracellular Water (ECW) Intracellular Water (ICW)

*ECG* electrocardiogram, *ICG* impedance cardiography

### Impedance Cardiography (ICG)

The Non-Invasive Continuous Cardiac Output Monitor (NICCOMO, Medis Medizinische Messtechnik GmbH, Ilmenau, Germany) was used for standardized automated sphygmomanometric blood pressure measurement on the right arm and with an appropriate cuff width. Impedance cardiography was performed using four electrodes (two on the axillary line under the thorax and two in the neck) eliminating skin resistance. The examination was performed after stabilization of cardiovascular function in standing position. Parameters were classified as blood pressures [diastolic (DBP), mean arterial pressure (MAP)], flow parameters [heart rate (HR), stroke volume (SV), cardiac output (CO)], contractility parameters [pre-ejection period (PEP), left ventricular ejection time (LVET), velocity index (VI), acceleration index (ACI)] and vascular parameters [total peripheral resistance (TPR)]. The latter was calculated using the formula (MAP × 80) / CO [15, 16].

### ECG-Doppler ultrasound

An electrocardiogram was combined with Doppler ultrasonography of the maternal renal interlobar veins, hepatic veins and the arcuate uterine arteries using a 3.5 MHz transabdominal probe during interrupted breathing in supine position (Aplio Mx, Toshiba Medical Systems nv, Sint-Stevens-Woluwe, Belgium). Each parameter was measured three consecutive times and averaged, reducing intra-variability [17]. Parameters of arteries and veins were classified into 2 groups: pulse transits and impedance indices.

The heart rate corrected venous pulse transit (VPT) is the time interval between the P-top from the ECG-wave and the A-wave from the Doppler pulse wave (PA in ms). In the arteries (heart rate corrected arterial pulse transit, APT), the time interval starts at the Q-wave on the ECG and ends at the start of the Doppler end-diastolic point D (QD in ms). The pulse transits are adjusted for heart rate, which is variable due to advancing gestation, and thereby divided by RR (time interval in ms between two consecutive R-waves of the ECG signal) [18].

At the venous side, the maximum and minimum flow velocity is measured from the renal and hepatic Doppler signal. An impedance index is calculated using the formula [(Maximum Velocity-Minimum Velocity)/Maximum velocity] [13, 19]. This renal interlobar vein index (RIVI) and hepatic vein index (HVI) are considered the venous equivalents of the arterial Resistivity Index (RI) which is calculated by the formula (Peak systolic velocity – End diastolic velocity)/Peak systolic velocity. In the uterine arcuate arteries, RI and Pulsatility Index (PI, (Peak systolic velocity – minimal diastolic velocity)/Mean velocity) were measured as reported [20, 21].

A combination of TPR and APT in dyn.sec/cm^2^, was also calculated and describes the relation between vascular resistance in the systemic vs. the uterine circulation (uterine flow promoting peripheral resistance (UFPPR)).

### Bio-impedance

The body composition and fluid balance were measured by a multiple frequency bioelectrical impedance analyzer (Maltron Bioscan 920-II, Maltron International LTD, Essex, UK) in supine position with stretched arms and legs, without socks or shoes [22]. Two electrodes, receiving the electrical signal, were placed on the dorsal surfaces of the wrist and ankle at the level of the process of the radial and ulnar resp. fibular and tibial bones. Two other electrodes, sending the electrical signal, were attached to the third metacarpal bone of the right hand and right foot. The applied current was 0,6 mA with a frequency of 5, 50, 100 and 200 kHz during 5 s. Total Body Water (TBW) was estimated, as the total of intracellular water (ICW) and extracellular water (ECW).

### Statistics

An independent t-test at 5% significance level was used for intergroup comparison of continuous demographic data. Chi-square test was used for categorical demographic variables. Normality was checked via Shapiro-Wilk. Data were presented as mean ± SD or n (%). These analyses were done in SPSS (SPSS Inc., Chicago, Illinois, USA).

The preliminary prediction models were calculated using a Partial Least Square Discriminant Analysis (PLS-DA), which discriminates NP from GH, NP from EPE and NP from LPE. All measured biophysical parameters were converted first to multiples of the expected normal median (MoM) per trimester. The PLS-DA evaluated the performance using (A) demographic data only; (B) demographic data + the maternal biophysical parameters at 12 weeks and (C) demographic data + the maternal biophysical parameters at 12 and 20 weeks. All pregnancies were used in the training set and cross-validation was used to quantify the performance. The number of observations in the training set decreased substantially when using multiple sets of biophysical parameters (ultrasound, impedance cardiography and bio-impedance variables) and data from both trimesters, due to missing data in one of the sets. Sensitivities, specificities, positive predictive values, negative predictive values, receiver operating curves (ROC) and likelihood ratios presented in this manuscript, were obtained with the PLS-DA with cross validation. SAS (SAS 9.4, Institute Inc., Cary, NC USA) was used for this data analysis.

## Results

A total of 969 pregnant women were measured at their first ultrasound appointment. Eight were categorized as EPE, 29 LPE, 35 GH and 897 NP. Patient and outcome characteristics of the four outcome groups are shown in Table 2.

**Table 2:** Patient and outcome characteristics in the four outcome groups

	Normotensive Pregnancy (NP) *(n = 897)*	Early-onset Preeclampsia (EPE) *(n = 8)*	Late-onset Preeclampsia (LPE) (*n* = 29)	Gestational Hypertension (GH) (*n* = 35)
Characteristics at inclusion				
Maternal age, years	30 (27–33)	30 (25–36)	30 (27–34)	30 (27–33)
Pregestational maternal weight, kg	64 (58–74)	76 (64–84)*	66 (62–82)	65 (59–79)
Pregestational maternal height, cm	166 (161–170)	166 (163–170)	166 (165–170)	167 (162–170)
Pregestational BMI, kg/m^2^	23,3 (21–26,8)	27,3 (23,3-29,8)	24,1 (22,4-30,2)	23,6 (22–27,9)
Gestational age at assessment, weeks+days				
First trimester	12w2d (11w5d-12w5d)	13w (12w3d-13w2d)*	11w5d (11w1d-12w5d)*	12w2d (11w6d-12w5d)
Second trimester	20w2d (19w6d-20w5d)	21w (20w3d-26w3d)*	20w5d (20w1d-21w4d)*	20w2d (19w5d-20w6d)
Medical history				
Diabetes Mellitus	116 (12,9%)	2 (25%)	6 (20,7%)	6 (17,1%)
Thrombophilia	33 (3,7%)	1 (12,5%)	1 (3,4%)	2 (5,7%)
Allergy	177 (19,7%)	0 (0%)	4 (13,8%)	13 (37,1%)*
Obstetric history				
Intra-Uterine Death	15 (1,7%)	0 (0%)	1 (3,4%)	1 (2,9%)
Intra-Uterine Growth Restriction	7 (0,8%)	0 (0%)	0 (0%)	1 (2,9%)
Nulliparity	472 (52,6%)	4 (50%)	8 (27,6%)*	14 (40%)
Hypertension				
Family history	41 (4,6%)	1 (12,5%)	1 (3,4%)	2 (5,7%)
Previous pregnancy	55 (6,1%)	2 (25%)	4 (13,8%)	10 (28,6%)*
Outcome characteristics				
Birth weight, g	3425 (3145–3734)	1300 (572–1712)*	3230 (2690–3656)*	3405 (3040–3720)
Birth weight, percentile	57,5 (33–77,5)	7,5 (3,8-48,8)	63,8 (28,1-79,4)	52,5 (23,5-82,5)
Gestational age at delivery, weeks + days	39w5d (38w5d-40w3d)	31w5d (27w-32w5d)*	37w5d (36w5d-39w6d)*	39w3d (38w5d-40w5d)

Data are presented as median (IQR) or n (%)

*p<0,05 was considered significant different from normotensive pregnancy (NP)

**Fig. 1:**
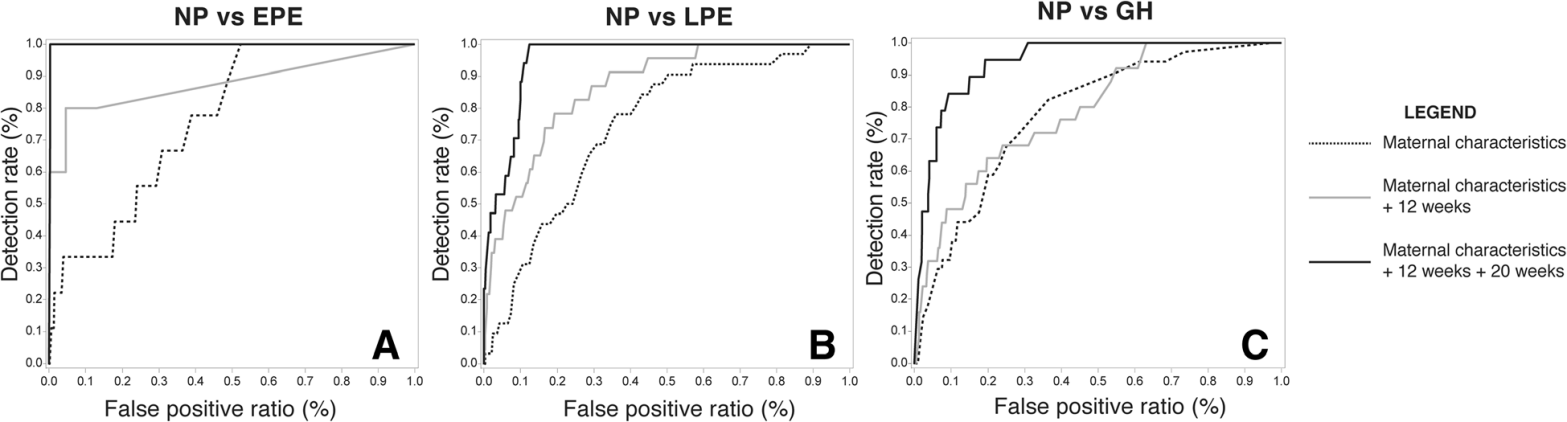
Receiving operating characteristics (ROC) curves of maternal characteristics, in stepwise combination with first and/or second trimester maternal biophysical parameters for the predication of early-onset preeclampsia (**a**), late-onset preeclampsia (**b**) and gestational hypertension (**c**). NP (Normotensive pregnancy); EPE (early-onset preeclampsia); LPE (late-onset preeclampsia); GH (gestational hypertension)

The best performance was obtained by combining the maternal biophysical parameters of first and second trimester together with the maternal characteristics. More specifically this means, the models include a set of demographical variables: maternal age, allergy, parity, length, BMI, history of hypertension/thrombophilia/IUGR/intra-uterine death/diabetes, and three sets of biophysical parameters (presented in Table 1), measured in first and second trimester (Fig. 1). Positive likelihood ratios (LR) are between 7,27–7,40 and the negative between 0 and 0,18.

### Early preeclampsia

The detection rate (DR) of EPE is 100% with false positive ratio (FPR) of 0%. Positive predictive value (PPV) is 67% and negative predictive value (NPV) is 100%. The area under the curve (AUC) is 99,8% (Fig. 1a).

### Late preeclampsia

At 14% FPR, the detection rate is 100%. The PPV is 21% and NPV 100%. The AUC for LPE is 94% (Fig. 1b).

### Gestational hypertension

The GH model is characterized by a detection rate of 84% at 12% FPR. This provides a PPV of 23% and NPV of 99%. The AUC is 95,3% (Fig. 1c).

## Discussion

This study is the first to demonstrate the feasibility of screening for all types of GHD using maternal biophysical parameters in combination with demographic parameters. Even without inclusion of biochemical parameters, our prediction models already show good performances (AUC of > 94%, DR of > 84%), but it is expected that these will improve further by combining these biophysical parameters with biochemical parameters. Validation in a large prospective study is now needed to evaluate the potential and applicability of biophysical parameters in screening for GHD.

Our study is the first to assess all major components of the maternal circulation as one integrated functional circle: volumes, heart, arterial and venous hemodynamics and are used as fundaments in a preliminary prediction model. A standardized protocol of non-invasive techniques with reported inter- and intra-observer correlations is used [12–14]. The bio-impedance technique may be criticized as being less valid than maternal echocardiography or dye dilution plasma volume measurements, however our results are in line with these so- called gold standard methods [23] and with other reports [24]. It should be appreciated that, similar to other reported methods [25], the bio- impedance methodology is very easy to perform with very low inter- and intra-observer variabilities, allowing a general application with a minimum of training or expertise. The total cardiovascular protocol is cheap and time efficient (15 min). We acknowledge that all pathological cases were rather low in this study and that EPE was based on very few measurements, even with inclusion of SGA and non-SGA, which might bias the model of EPE. The statistics behind the models is also still too complex and preliminary to provide us already with a defined algorithm with corresponding coefficients. Another point of attention is that 13.5% of our studied population has diabetes mellitus and uses insulin for therapy with potential interference with our cardiovascular measurements.

Current guidelines on screening and management of women at high risk for hypertension in pregnancy are based on maternal risk factors only, and performance is rather low with 35% detection rate for preeclampsia and 40% for early preeclampsia [26, 27]. In practice, diagnosing GHD is based on elevated blood pressures and/or proteinuria during a routine clinical visit in late second or third trimester, and at that stage, disease is already established and preventive therapy is not useful anymore. Therefore, various models were investigated to improve first trimester prediction. The current best approach combines maternal characteristics with first trimester uterine artery Doppler pulsatility index, MAP and a combination of biomarkers (PlGF, sFlt-1 or PAPP-A), reaching detection rates of > 90% for EPE and between 35 and 76% for LPE by a 10% FPR [3–5]. This means EPE has a good detection rate, but performance on LPE is poor [3–5, 26]. Prediction of GH scores even lower (18–21% at 5% FPR) [5]. Compared to those latter models, the prediction models in our study promise better (preliminary) detection rates on all diagnoses.

In multiple studies [10], an extended list of pathologic biophysical parameters are measured already in the first trimester: higher uterine pulsatility indices, higher TPR, lower ACI and VI, higher CO and SV, etc. We have shown before that type-specific cardiovascular characteristics allow discrimination between GH, EPE and LPE [11, 21]. More and more studies use maternal hemodynamics to explain the pathophysiology of preeclampsia and other hypertensive disorders. It is important to focus not only on (early) preeclampsia, but on all types of hypertension when creating a prediction tool. Therefore, we aimed to explore a different fundament for a prediction model than the current popular research tracks, and focus on all hypertensive cases.

The models in our study showed the best performance when adding the first and second trimester parameters together. Other prediction models use only first trimester information. However, it is shown that all women with GHD have a gradual worsening in cardiovascular dysfunction [11]. It is therefore not illogical to introduce the second trimester parameters into a prediction model. Women at risk could be considered for preventive treatment such as low-dose aspirin depending on the grade of cardiovascular maladaptation and the predicted first trimester risk profile [28]. But adding a second trimester assessment could confirm more accurately the risk status and determine a personalized follow up monitoring, such as remote monitoring [29–31] and/or tailored hemodynamic-based antihypertensive therapy [32].

Gestational hypertensive disorders are not only linked to cardiovascular maladaptations throughout a pregnancy, but predisposes also to cardiovascular diseases in later life, including a potential hypertensive risk in a future pregnancy [33, 34]. Understanding completely the behaviour of the cardiovascular system before, during and after a pregnancy together with the knowledge of correct measuring these biophysical parameters, could contribute to the advancement of the current approach of gestational hypertensive disorders. It is important to screen patients in time to possibly prevent or at least delay the hypertensive problem.

These first preliminary results of our approach look very promising, but further research is absolutely necessary to validate the real performance. It offers a possible new way of solving the limitations in the currently investigated prediction models based on biochemical parameters. Reported incidence of hypertensive disorders of pregnancy varies between countries, ranging between 1.4–4.0% for preeclampsia overall, 0.3–0.7% for early onset preeclampsia and 3.6–9.1% for gestational hypertension [35]. Incidences are very similar in our study population, with 3.8% (37/969) for preeclampsia overall, 0.8% (8/969) for early onset preeclampsia and 3.6% (35/969) for gestational hypertension respectively. As such, our study population could be considered representative for a general obstetric population, however it is to be recognized that we excluded pregnancies complicated with EH, superimposed late preeclampsia with or without SGA, HELLP with or without SGA, isolated SGA and multiplets from the prediction model analysis. Next to this, the Achilles’ heel of our screening method is maternal venous Doppler sonography, which not only is subject to human error but also vulnerable for a variety of interfering anatomo-physiologic factors such as respiration, muscle tension, anatomical variability, etc.… [36]. High inter- and intra-observer variability is reported for maternal venous Doppler measurements, markedly improving to intraclass correlations > 0.9 with the use of (a) an ECG signal as a reference for Doppler wave interpretation, (b) repeated measures and (c) sonographer’s training [17]. As such, the performance of our screening algorithm in a general population is to be explored independently in a new prospective assessment, with consideration of sonographer’s training, performance and availability, of patient’s access and costs, of audit and feedback mechanisms and of impact on overall population health and public costs [37, 38]. The presented prediction models are therefore certainly not ready yet for use in clinical practice in its current form. This paper inspires hopefully other clinicians and researchers to investigate the first and/or second trimester maternal hemodynamics and its prediction potential.

## Conclusions

This is the first study that uses the cardiovascular physiology as fundament for a screening model for hypertensive disorders. It includes only biophysical and demographic parameters, showing prudently good performances (AUC > 94%). This study is very topical, because there is an urgent need for a good performing screening model to identify not only the preeclamptic population, but any patient who could suffer from hypertension. Experiencing hypertension in a pregnancy is a forerunner of a bad cardiovascular health in later life. Preventing or delaying it is of utmost importance. This study might bring a potential new and fresh input in the current research for screening models.

## Figures and Tables

**Fig. 2:**
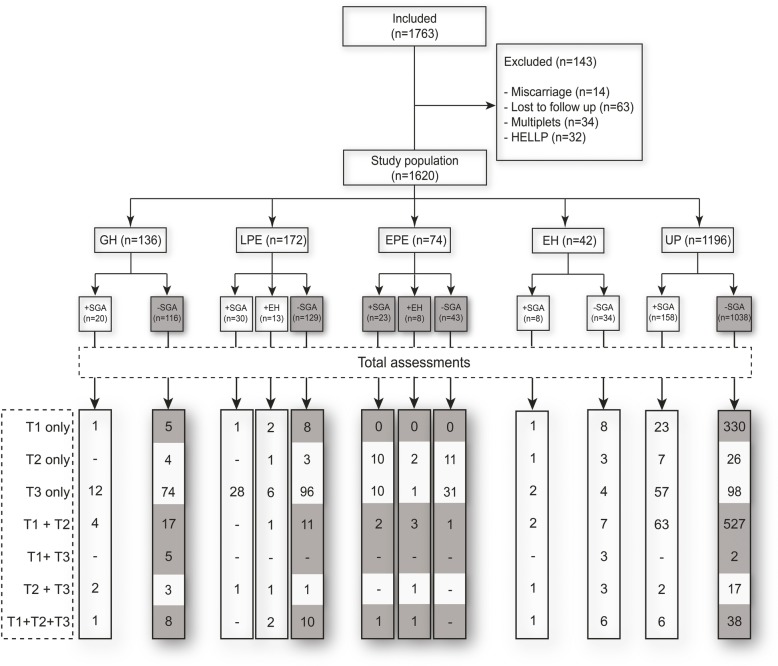
Flowchart summarizing the total numbers of patients and assessments in each trimester, obtained from the observational study between 2011 and 2016 as part of the Hasselt University Research Project of Maternal Venous Hemodynamics. The grey zones are the patients included in this analysis. *T1: trimester 1; T2: trimester 2; T3: trimester 3; GH: Gestational hypertension; LPE: Late preeclampsia; EPE: Early preeclampsia; EH: Essential hypertension; SGA: Small for gestational age*

## Abbreviations

ACI: Acceleration index
APT: Arterial pulse transit
AUC: Area under the curve
BMI: Body mass index
CO: Cardiac output
DBP: Diastolic Blood Pressure
DR: Detection ratio
ECW: Extracellular water
EH: Essential hypertension
EPE: Early preeclampsia
FPR: False positive ratio
GH: Gestational hypertension
GHD: Gestational hypertensive disorders
HR: Heart rate
HVI: Hepatic vein index
ICW: Intracellular water
LPE: Late preeclampsia
LR: Likelihood ratios
LVET: Left Ventricular Ejection Time
MAP: Mean arterial pressure
MoM: Multiples of expected normal median
NICCOMO: Non-Invasive Continuous Cardiac Output Monitor
NP: Normotensive pregnancy
NPV: Negative predictive value
PAPP-A: Pregnancy-associated plasma protein A
PEP: Pre-ejection period
PI: Pulsatility index
PlGF: placental growth factor
PLS-DA: Partial Least Square Discriminant Analysis
PPV: Positive predictive value
RI: Resistivity Index
RIVI: Renal interlobar vein index
ROC: Receiver operating curves
sFlt-1: Soluble FMS-like tyrosine kinase-1
SGA: Small for gestational age
SV: Stroke volume
TBW: Total Body Water
TPR: Total peripheral resistance
UFPPR: Uterine flow promoting peripheral resistance
VI: Velocity index
VPT: Venous pulse transit
